# Empathy is proprioceptive: the bodily fundament of empathy – a philosophical contribution to medical education

**DOI:** 10.1186/s12909-018-1161-y

**Published:** 2018-04-05

**Authors:** Florian Schmidsberger, Henriette Löffler-Stastka

**Affiliations:** 1 Institute for Philosophy, and University Program for Psychotherapeutic Propedeutics, Postgraduate Center, University of Vienna, Vienna, AT Austria; 2 Department for Psychoanalysis and Psychotherapy, University Program for Psychotherapy Research, Teaching Center, Medical University of Vienna, Postgraduate Center, University Vienna, Währingerstraße 18-20, 1090 Vienna, Austria

**Keywords:** Empathy, Medical education, Training, Phenomenology of emotions, Philosophy, Simulation, Interaction, Thomas Fuchs

## Abstract

**Background:**

The current philosophical debate on empathy entails accounts of theory of mind and simulation as well as a phenomenological opposition. The first focuses on a detached observation of others from a 3rd person perspective and formulates the common claim that there is no direct access to the mental and emotional life of others, only simulation or analogy can grant access to the emotions and behaviour of others. The philosophical respectively phenomenological account of Fuchs instead opposes by focusing personal interaction within a 1st or 2nd person perspective claiming that the emotions of others are experienceable through bodily expression and bodily resonance. Fuchs offers an account of embodied affectivity that emphasizes the role of the (subjective) body for emotion and empathy. By experiencing the bodily expressed emotions of a vis-à-vis with and through the own body empathy and social understanding are bodily grounded. Following this core thesis Fuchs differentiates a primary, bodily empathy and an extended empathy that focuses on putting myself in the shoes of others (perspective taking).

**Discussion:**

By comparison of different forms of social understanding as discussed in the phenomenological tradition – like contagion, sharing and empathy – it can be shown that extended empathy has an egocentric character. By putting myself in the shoes of others I miss a person’s otherness that transcends my capacity of imagination respectively the personal frame of my experience. Further Fuchs’ disregards that a bodily based empathy is co-structured by higher level form of understanding like contextual biographic knowledge.

**Conclusion:**

The philosophical discussion offers fertile impulses for Medical Education (ME) and the training of empathic communication skills. The account of Fuchs highlights the role of bodily perception (proprioception) as a resource of understanding others. Thus proprioceptive skills of a physician can support the empathic understanding of the physician. The objection against the egocentric trait of perspective taking admonishes not to generalize the own perspective as decisive for empathy and to adopt an attitude that remains open to the otherness of a patient and its experiences.

## Background

Empathy is of special relevance to the relationship of the clinician and the patient. It fosters the satisfaction of the patient, supports a better compliance and therefore has an impact on the clinical outcome. An empathetic communication with the patient is in opposition to a paternalistic prescription of a course of treatment [1]. Thus it is not astonishing medical education (ME) focuses on the training of empathy. Every tuition of empathic skills is based upon a concept of empathy. The aim of this paper is to reflect on a contemporary notion of empathy from a philosophical point of view. It will focus on the recent phenomenological account of the physician and philosopher Thomas Fuchs.

### Philosophical discourse: simulation, theory of mind and phenomenological opposition

The current philosophical debate on empathy entails accounts of theory of mind and simulation as well as a phenomenological opposition [2–5]. While a *theory of mind theory* considers social understanding as based upon an innate or acquired theory of behaviour and motives respectively on inferences about other’s mental states, *simulation theory* sees the use of our own mind to simulate the mental states of others – as if we were in their situations – as the paradigm for social understanding, an understanding of the beliefs, desires and emotions of others. As a consequence empathy is fundamentally based »on mind-reading or on simulating others’ mental states inside oneself« [6]. Despite their differences they articulate the common approach to social understanding within a third person paradigm, a passive and detached observation of other’s behaviour, neglecting a participating interaction with others [7]. They also share the common claim that it is impossible »to *experience* other minds, both presuppose the fundamental opacity or invisibility of other minds« [8]. In contrast phenomenological accounts offer an approach to social understanding and empathy upon an embodied interaction within a practical and shared context, granting an access to other minds or the emotional life of others through visible and experienceable bodily expressions – like Thomas Fuchs.

Fuchs’ descriptions of empathy are based on an account of embodiment. According to this approach mind and body may not be understood as separated and isolated but as intertwined. Instead of speaking of a body in contrast to the mind (or vice versa) the account of embodiment favours the terms of a »minded body« or an »embodied mind« [9], understanding our experiences as »formed and influenced by our embodiment« [10]. On the one hand an account of embodiment is deeply rooted in philosophical concepts of the body, namely of the phenomenological tradition (especially Merleau-Ponty); on the other hand it applies in innumerable empirical studies researching the intertwinement of body and mind. For example, the consequence of hunger for juridical judgments [11] or the influence of our bodily posture for positive or negative memories [12].

For the discussion of empathy embodiment has the consequence that the emotional and cognitive life of a vis-à-vis is principally accessible throughout his visible bodily expression and mutual incorporation. For Fuchs a bodily face-to-face encounter is fundamental and prior. His approach can generally be assigned to an interactionist account [13], focussing a »joint engagement« and »shared involvement in social event [sic!]« with a »co-precence« or »being with one another« [14]. Interaction theory accounts replace a solipsistic internal simulation of others by a mutual bodily joint encounter.

### Embodied affectivity: being bodily directed towards

The account of Fuchs is based upon the traditional phenomenological distinction between subject body (Leib) and object body (Körper). The subject body is the perceiving and subjectively lived body, the object body is the perceived body [15]. This differentiation can already be instructive for physicians: when a patient enters the room and the physician talks to him, how she feels and what’s her issue to consult the physician, she is dealing with a subject, with a person. By offering one’s hand to give a handshake of the patient, the patient’s body is perceived as a subject body, the person is in the foreground. When the physician for example takes an instrument to investigate the eyeground a process of »zooming in« respectively a change of attitude (»Auffassungsänderung« [16]) takes place. The person steps into the background, the physical body steps into the foreground, microprocesses of the person are focused while the subject aspects of the person vanish. This basic phenomenological differentiation helps to understand that *the human body has different appearances according to the attitude and the perspective of the physician*. The subject attitude helps to understand the subject issues of a patient and conceals the microprocesses of the object body; while otherwise the object attitude discovers those bodily microprocesses while concealing the subjective aspects of a human life.

The understanding of that basic philosophic differentiation can be deepened by carrying out the following exercise – we recommend medical students to perform this to make a personal experience with the named difference: Sit patiently with some spatial distance next to another person, look at her eyes and get immersed in the interplay of your gazes. You also can focus an object like a filled bottle or a lamp. When doing so where do your physical and your subjective body end? – When performing this you will probably make the experience of an ambiguity: your physical body ends at the position where you are sitting, while on the other hand your attention and your gaze will extend »into« the room of the concrete situation of your performance. You don’t receive your vis-à-vis or the focused object as pictures in your brain but more likely as »objects« »in the world«, your subject body extends into the world, maybe getting intertwined in the play of gazes with your vis-à-vis. Your subjective body somehow transcends your physical body making the different appearance of a human body experienceable.

Within his philosophical elaborations Fuchs focuses on the subjective body. He considers the body as »co-constitutive« [17] for the cognitive and emotional life. By emphasizing their embodiment he opposes to approaches that accentuate the cognitive or evaluate contents of emotions and that disregard the role of the body.

Body and emotions belong together very closely. His concept of an embodied affectivity entails an *affective intentionality* and a *bodily resonance*. Affective intentionality means that emotions are directed towards objects, persons or situations, discovering, what is of meaning and value for us. *Bodily resonance* means that everything that is experienced is experienced *through and with the body.* This entails autonomous nerve activities (e.g. heartbeat, breath, tremor), muscular activities, bodily postures or gestures. Especially the face, the chest and the stomach may be seen as »[p]articularly rich fields of bodily resonance.« [17] Through and with our body we are directed towards objects, persons and situation in our emotions: »Being afraid, for instance, is not possible without feeling a bodily tension or trembling, a beating of the heart or a shortness of breath, and a tendency to withdraw. It is *through* these sensations that we are anxiously directed toward a frightening situation.« (ibid.) Against this background it may not be very surprising that the body has a principal medial character: the body serves as the »medium of our affective engagement in a given situation« [18]. This »mediality« may be best understood by the formulation: »*with and through* the body«.

### Intercorporality and interaffectivity

The central point and the essential presupposition for this approach is that *emotions are visible through our bodily (or bodily mediated) expression*: »[emotions] are not only felt from the inside, but also displayed and visible in expression and behaviour« [19]. This is opening a field that Fuchs is calling »intercorporeality« and »inter-affectivity«, where the bodies of several persons are intertwined (»bodily link« [20]) and an »embodied communication« [21] is enabled.

Fuchs is illustrating that with the example of anger: two persons are having a dispute, the first person turns angry and expresses it through the things she says, an aroused voice, a blushed face and an offensive posture. The second person is confronted with the bodily expression of her counterpart experiencing it bodily too: she feels a displeasing tension and an impulse to withdraw. The expression of person A becomes the impression for person B. The body of the second person incorporates the first person, the perception of the other is mediated by her own body. The expression of the first person turns into the impression for the second person that responds bodily herself for example with an intimidated posture. The expression of the second person becomes an impression for the first person – and so on. In such a way a *process of inter-affectivity*, a circular process of impressions and expressions that follow each other emerge. It is about a process that takes place within split seconds on a sub-conscious level where the bodies of the involved persons get intertwined. Fuchs is calling such a process »mutual incorporation« [21] what means, that the emotion of the other is *sensed and experienced with the own body through bodily resonance* [22].

Within such processes of bodily intertwinement Fuchs finds »the bodily basis of empathy and social understanding« [23] respectively »kinaesthetic empathy« [22]. From his perspective empathy and social understanding are not based upon reflective and cognitive efforts of inferring, decoding or deliberately simulating other minds, but on a pre-reflective, bodily and non-verbal interaction. In such a way the own body also becomes a medium of the participation on a common sphere [24], it becomes a »*medium* of inter-affectivity and empathic understanding« [25].

### Levels of empathy: primary and extended empathy

With his younger, not yet published works, Fuchs offers an important differentiation for describing empathy. He distinguishes different levels of empathy: a »primary empathy« [26] respectively a »basic empathy« [27] that is constituted thorough mutual bodily incorporation and interaction and »higher-level forms of social understanding« [28] or »extended empathy« [29], such as perspective taking or »imaginary transposition« [27], i.e. that I imagine how it would be for me to be in someone’s shoes.

The first one, »primary« or »basic empathy« was already sketched in the passages before: Emotions of others are sensed with the own body through bodily resonance – an »empathic perception« [30]. What is understood by that? It is an immediate understanding in which emotion someone is currently immersed, what emotional state is recently expressed bodily within the common situation*.* Such intercorporeal understanding emerges from a common history that starts in the early days of our lives: »infants are already able to perceive the emotions and intentions in the actions of others in their posture, gestures and facial expressions, as related to the context of the common situation.« [28] Within such references Fuchs elaborates the concepts of intercorporeal memory and a habitus that arises from infant interaction [31]. So the bodily basis of empathy points to a history with close attachment figures within practical contexts.

The level of extended empathy refers to the phenomena that simulation theory points at: putting oneself in the shoes of others. Fuchs concedes that primary empathy »does not exhaust the possibilities of empathic understanding and intersubjectivity« [28]. In addition he names non pre-reflective, conscious and deliberate forms like an explicit representation of someone or imagination of someone’s situation, perspective taking, imaginative transposition [29], inferring to hidden intentions, language or narratives. Such complex forms of empathy may be consulted when irritations, misunderstandings, distortions occur, when we for example don’t understand why someone reacted so aroused and angry. Fuchs describes such forms of empathy as an »explicit, cognitive operation«, an »imaginative operation«, a »transposition into an ‘as if’ scenario«, a deliberate form instead of an »involuntary coupling of mutual incorporation« [29]. By that the bodily aspects may step in the background, it »transcends the bodily level« [29] of primary empathy.

But simulation theory has a »(limited) justification« [26]. The limitation means that this form of empathy may not be seen as the key- and leading mode of empathy: »Simulation theory incorrectly generalizes the possibility of imaginative transposition or simulation [...] to all kinds of empathy« [25]. For understanding the anger of a person in an immediate encounter, this form of empathy is neither necessary, nor sufficient – primary empathy remains prior for Fuchs.

## Discussion

### Relations of empathy: different forms of social understanding

We want to continue by carving out different constitutive relations within different forms of social understanding, referring to other approaches of the phenomenological tradition on empathy [2]. Three forms can be distinguished: *contagion, sharing* and *empathy*. *Contagion* means that you are infected with a feeling through an atmosphere, without knowing what the concrete object of the emotion is [32]. For example you come late for a general meeting of a certain society. When entering the room you feel an aggressive atmosphere though you don’t know whether there is a conflict currently going on or what it is about. Still you turn into an aggressive and offensive mood yourself.

If you are in the mode of *sharing* you are directed towards the same object as another person experiencing the same emotion as the other. For example when a friend tells you about the relationship problems with her partner and you feel the same anger about her partner as your friend. You put yourself in the shoes of your friend and consider how it would be for you to be in that situation. There is a trait about this form of social understanding that also can be considered as a fundamental objection against a simulational account: sharing is based upon a perspective taking where I put myself in the shoes of others followed by an analogy where I project my emotions onto the other. Thus sharing can be seen as a reiteration of myself. And what do I understand with that? »I understand only myself in that other situation, but I don’t necessarily understand the other.« [33] So this kind of social understanding has an *egocentric trait*. By this form you wouldn’t be able to grasp the ways how others are feeling differently to you. Especially in medicine one is often confronted with persons and ways of feeling one couldn’t even think of. So this form of social understanding remains blind for the alien and the otherness of others that transcends the capacity of the own imagination.

In *empathy* you are instead the object of your emotion is different to the empathized person. For example when a parent is mourning her deceased child, the parent is directed at the dead child. Within empathy you are not directed at the dead child but at the grief of the parent, feeling compassion and pity for the parent. So empathy has essentially *another object* as sharing and brings you into a *different emotional* state than the person you are empathizing with. This difference is constitutive, there is a »self-other-differentiation« respectively a gap between my experience and the experience of the other. The differentiation means: »In empathy, the experience you empathically understand remains that of the other. The focus is on the other, and not on yourself, not on how it would be like for you to be in the shoes of the other. That is, the distance between self and other is preserved and upheld.« [32] While sharing has an *egocentric trait* empathy instead has an *allocentric* trait.

Against this background it is to criticise that within »extended empathy« Fuchs offers more a relation of sharing than a notion of empathy, we even would assert that Fuchs misses the phenomenon of empathy on that level. By referring to »putting oneself in the shoes of another« [29] he follows the *egocentric* account of simulation theory. Imaginary transposition doesn’t get the alien and the otherness of the other’s perspective that may often transcend the own imagination. It simply doesn’t get the phenomenon that others may feel in ways one wouldn’t even think of – e.g. when a patient deliberately harms himself by pushing 12 needles of an insulin syringe below the kneecap of his right leg.

But how to access and to experience this foreign otherness in the emotions of a vis-à-vis? It is accessible through primary empathy and on one’s own bodily resonances when the other person tells her story and expresses her feelings. As drawn before such encounter is more than an imaginary transposition, it means to feel the expressed experience of the others with and through the own body. A further decisive presupposition is the attitude of avoiding an egocentric imagination, how it would be for me within my frame of experience.

### Levels of empathy: bodily interaction and mediated contextual knowledge

Further the »extended empathy« of Fuchs has to be criticised as it misses *mediated contextual knowledge* that is more than perspective taking. It is the claim that extended empathy is more complex than drawn by Fuchs. Mediated contextual knowledge of a person and general knowledge of human behaviour transcends the situation of the interaction. It is a more comprehensive knowledge about the world of meaning of the other that may be acquired through dialog and common conversation (instead of observation) with another. While intercorporeality and mutual incorporation proceed involuntarily, this form of social understanding is more voluntarily. This understanding of another person is more complex, it entails an understanding of beliefs, motives, a biographic situation and biographic history. It is an understanding of a person that one acquires over a longer period of acquaintance (e.g. through psychotherapy or friendship). It relies on interpretation and on »highly structured contexts of meaning« [34] where the observation of expressive movements and action do not suffice [34] and where embodiment steps into the background.

This can be illustrated by a psychiatric patient who suffers from paranoid schizophrenia, showing bizarre ideas in delusions. Psychotherapeutic work aims at making sense of those ideas by discovering the subjective meanings of the delusions that may become plausible against the background of the patient’s biographic situation. The delusion of the patient may be empathically understood not just by her bodily expressions in a concrete common situation of the dialogue unit but through contextual knowledge that transcends the situation, a present pre-reflective knowledge of the patient’s biographical circumstances. This contextual knowledge supports the professional empathy for the emotion the patient is dealing with. In that way the contextual knowledge even influences the bodily resonance of the professional, as it furthers his sensitivity for the emotions of the patient.

Zahavi [35] points out that the direct bodily encounter and the indirect, referring and contextual understanding are co-present during an interaction with others. Especially with examples from psychotherapeutic practice I want to claim that mediated contextual knowledge influences immediate bodily resonance. That means that Fuchs’ »bodily basis of empathy« *is co-constituted and co-structured by higher-level forms of knowing and understanding others that are not pre-reflective*.

## Conclusion

What are the benefits of such philosophic elaborations for the professional practice of a physician or for ME? First the phenomenological account of Fuchs emphasizes a bodily basis of empathy, claiming that the own bodily resonance towards the expressed emotion of a vis-à-vis is prior and fundamental to empathetic understanding. For ME and the tuition of empathy skills this means to put an additional focus the bodily resonances of the physician when talking to a patient as it can be seen as a sensory for his emotions. Proprioception and proprioceptive skills become elementary for empathy and the communication with the patient.

This can be trained by exercises like the following: Watch the Oscar awarded documentary »Journey into self« with Carl Rogers and Richard Farson from 1969, focus on one of the shown stories, for example on the story of the man who doesn’t want to have any friends and breaks out into tears when talking about it, and observe your own bodily resonances when watching the sequence. The different personal bodily experiences can further be reflected within the group of students guided by a professional. The reflection aims at fostering proprioceptive awareness, understanding the personal meaning of such personal bodily occurrences, comparing it to the bodily experiences of others, understanding commonalities and differences to colleagues and also to reflect on the meta-level that the own subject body grants an access to the emotion of others. If your medical training also encompasses training sequences with actors the bodily resonance of the students during the concrete situated interaction can be subject of such reflection [36].

Second the discussion of his Fuchs’ account showed that within empathic understanding an immediate bodily resonance and a mediated contextual knowledge are co-present. It also pointed to the restriction of strategies of perspective taking, imaginary transposition or analogy: the procedure of putting yourself in the shoes of others and projecting your own experiences on the other has an egocentric trait that misses the experiences that are out of the range of your own capacity of imagination. For ME it is to point out that empathy needs both, the bodily resonance and the contextualized knowledge about the patient. It also emphasizes a decisive differentiation between self and other and to remain incredulous against the temptation to generalize the own imagination as the leading trait of empathy. Such an attitude can be trained by reflecting on the personal experiences (like drawn above) in the group with other students [1, 36]. Then students get confronted with a plurality of experiences, meaning that one interaction with a patient can be understood and bodily experienced in various way according to the involved persons and their perspectives respectively biographic backgrounds. This should help to relativize one’s own experience, to avoid taking your own stance as exclusive and to bring oneself out of centre of one’s own perspective.

## Abbreviations

ibid: ibidem
ME: Medical education

## References

[1] Löffler-Stastka H (2017). Empathy in psychoanalysis and medical education - what can we learn from each other?. BMC Med. Educ.

[2] Zahavi D (2010). Empathy, embodiment and interpersonal understanding: from Lipps to Schutz. Inquiry.

[3] Gallagher S (2012). The phenomenological mind.

[4] Slaby J (2014). Empathy's blind spot. Med. Health Care Philos.

[5] Fuchs T, Schlimme J (2009). Embodiment and psychopathology: a phenomenological perspective. Curr. Opin. Psychiatry.

[6] Fuchs T. Intercorporeality and Interaffectivity. In J. Meyer, J. Streeck, & S. Jordan. Intercorporeality: emerging Socialities in interaction. Oxford: Oxford University Press. To be published. p. 1.

[7] Fuchs T, De Jaegher H (2009). Enactive Intersubjectivity: participatory sense-making and mutual incorporation. Phenomenol Cogn Sci.

[8] Zahavi D (2010). Empathy, embodiment and interpersonal understanding: from Lipps to Schutz. Inquiry.

[9] Gallagher S (2012). The phenomenological mind.

[10] Gallagher S (2012). The phenomenological mind.

[11] Gallagher S (2012). The phenomenological mind.

[12] Fuchs T, Koch S (2014). Embodied affectivity: on moving and being moved. Front. Psychol.

[13] Slaby J (2014). Empathy’s blind spot. Med. Health Care Philos.

[14] Slaby J (2014). Empathy's blind spot. Med health care and. Philosophy.

[15] Fuchs T, Schlimme J (2009). Embodiment and psychopathology: a phenomenological perspective. Curr. Opin. Psychiatry.

[16] Waldenfels B, Waldenfels B (1980). Intentionalität und Kausalität. Der Spielraum des Verhaltens.

[17] Fuchs T, Koch S (2014). Embodied affectivity: on moving and being moved. Front. Psychol.

[18] Fuchs T, Koch S (2014). Embodied affectivity: on moving and being moved. Front. Psychol.

[19] Fuchs T, Koch S (2014). Embodied affectivity: on moving and being moved. Front. Psychol.

[20] Fuchs T, Koch S (2014). Embodied affectivity: on moving and being moved. Front. Psychol.

[21] Fuchs T, Koch S (2014). Embodied affectivity: on moving and being moved. Front. Psychol.

[22] Fuchs T. Levels of Empathy. In V. Lux, & S. Weigel, editors. Empathy: A neurobiologically based capacity and its cultural and conceptual history. Palgrave Macmillan; to be pulbished. p. 7.

[23] Fuchs T. Levels of Empathy. In V. Lux, & S. Weigel, editors. Empathy: A neurobiologically based capacity and its cultural and conceptual history. Palgrave Macmillan; to be pulbished. p. 5.

[24] Fuchs T (2013). Depression, Intercorporeality and Interaffectivity. J Conscious Stud.

[25] Fuchs T. Levels of Empathy. In V. Lux, & S. Weigel, editors. Empathy: A neurobiologically based capacity and its cultural and conceptual history. Palgrave Macmillan; to be pulbished. p. 13.

[26] Fuchs T. Levels of Empathy. In V. Lux, & S. Weigel, editors. Empathy: A neurobiologically based capacity and its cultural and conceptual history. Palgrave Macmillan; to be pulbished. p. 4.

[27] Fuchs T. Intercorporeality and Interaffectivity. In J. Meyer, J. Streeck, & S. Jordan. Intercorporeality: Emerging Socialities in Interaction. Oxford: Oxford University Press. To be published. p. 2.

[28] Fuchs T. Intercorporeality and Interaffectivity. In J. Meyer, J. Streeck, & S. Jordan. Intercorporeality: Emerging Socialities in Interaction. Oxford: Oxford University Press. To be published. p. 18.

[29] Fuchs T. Levels of Empathy. In V. Lux, & S. Weigel, editors. Empathy: A neurobiologically based capacity and its cultural and conceptual history. Palgrave Macmillan; to be pulbished. p. 11.

[30] Fuchs T. Levels of Empathy. In V. Lux, & S. Weigel, editors. Empathy: A neurobiologically based capacity and its cultural and conceptual history. Palgrave Macmillan; to be pulbished. p. 8.

[31] Gallagher S (2012). The phenomenological mind.

[32] Zahavi D (2010). Empathy, embodiment and interpersonal understanding: from Lipps to Schutz. Inquiry.

[33] Gallagher S (2012). The phenomenological mind.

[34] Gallagher S (2012). The phenomenological mind.

[35] Zahavi D (2010). Empathy, embodiment and interpersonal understanding: from Lipps to Schutz. Inquiry.

[36] Drdla S, Löffler-Stastka H (2016). Influence of conversation technique seminars on the doctoral therapeutic attitude in doctor-patient-communication. Wien Klin Wochenschr.

